# Levels of Sex Hormones and Abdominal Muscle Composition in Men from The Multi-Ethnic Study of Atherosclerosis

**DOI:** 10.1038/s41598-024-66948-4

**Published:** 2024-07-12

**Authors:** Amar Osmancevic, Matthew Allison, Iva Miljkovic, Chantal A. Vella, Pamela Ouyang, Penelope Trimpou, Bledar Daka

**Affiliations:** 1 General Practice / Family Medicine, School of Public Health and Community Medicine, Institute of Medicine, Sahlgrenska Academy, University of Gothenburg, Gothenburg, Sweden; 2 Division of Preventive Medicine, School of Medicine, UC San Diego, San Diego, CA USA; 3 Department of Epidemiology, School of Public Health, University of Pittsburgh, Pittsburgh, PA USA; 4 Department of Movement Sciences, University of Idaho, Moscow, ID USA; 5 Institute for Clinical and Translational Research, Johns Hopkins University School of Medicine, Baltimore, MD USA; 6 Department of Endocrinology, Institute of Medicine, Sahlgrenska Academy, University of Gothenburg and Sahlgrenska University Hospital, Gothenburg, Sweden

**Keywords:** Obesity, Endocrine reproductive disorders, Ageing, Skeletal muscle, Epidemiology

## Abstract

Information on the associations of testosterone levels with abdominal muscle volume and density in men is limited, while the role of estradiol and SHBG on these muscle characteristics are unclear. Therefore, this study aimed to investigate the association between fasting serum sex hormones and CT-derived abdominal muscle area and radiodensity in adult men. Conducted as a cross sectional observational study using data from the Multi-Ethnic Study of Atherosclerosis, our analyses focused on a community-based sample of 907 men aged 45–84 years, with 878 men having complete data. CT scans of the abdomen were interrogated for muscle characteristics, and multivariable linear regressions were used to test the associations. After adjustment for relevant factors, higher levels of both total testosterone and estradiol were associated with higher abdominal muscle area (1.74, 0.1–3.4, and 1.84, 0.4–3.3, respectively). In the final analyses, levels of total testosterone showed a positive association, while an inverse relationship was observed for SHBG with abdominal muscle radiodensity (0.3, 0.0–0.6, and − 0.33, − 0.6 to − 0.1, respectively). Our results indicate a complex association between sex hormones and abdominal muscle characteristics in men. Specifically, total testosterone and estradiol were associated with abdominal muscle area, while only total testosterone was associated with muscle radiodensity and SHBG was inversely associated with muscle radiodensity.

*Clinical Trial*: NCT00005487

## Introduction

Abdominal obesity is linked to a higher risk of cardiometabolic disorders and mortality^1^. However, recent evidence suggests the quality and quantity of abdominal muscles may also play an important role in cardiometabolic health^2^. In this regard, abdominal muscle radiodensity, measured in Hounsfield Units (HU), reflects the function of muscle tissue and the degree of fat infiltration/fibrosis^3^. Of note, growing evidence suggests that abdominal muscle radiodensity is inversely associated with cardiovascular events and mortality in men^2^.

Earlier studies showed testosterone induces muscle fiber hypertrophy and increases the number of myonuclear cells by regulation of protein synthesis, satellite cells and stem cell proliferation, in addition to stimulation of the myogenic lineage and inhibition of the adipogenic cell line by activating androgen receptors (ARs)^4,5^. It is known that testosterone exerts regulatory control over pivotal proteins engaged in molecular processes such as glycolysis, glycogen synthesis as well as lipid and cholesterol metabolism^6^. Moreover, in men with testosterone deficiency, replacement therapy improves muscle volume, strength and quality of life^7^. However, it is unknown whether endogenous testosterone concentrations have different effects on different abdominal muscle functional groups. In addition, the effects of estradiol on muscle characteristics in men are unclear. In this regard, Russel and Colleagues reported in their review, no clear effect of estradiol on muscle mass or strength in men^8^. Yet, other studies have shown that estradiol could play a key role in regulating abdominal adiposity but is also directly associated with the volume of lean mass in men and appears to prevent expansion of adiposity^8–10^.

While there is some evidence to suggest that both testosterone and estradiol play an important role in the regulation of muscle function and volume, the nature of this relationship is not yet well established. Moreover, the bioactive role of sex hormone-binding globulin (SHBG) is still limited. Given this, we examined the cross-sectional associations between sex hormones and abdominal muscle characteristics in a large multi-ethnic sample of middle-aged and older men and hypothesized that higher estradiol and testosterone levels would be associated with greater abdominal muscle area, while testosterone would also be associated with higher abdominal muscle radiodensity.

## Results

Baseline characteristics of the study population are presented in Table 1. The mean age was 61.6 years. The majority of participants where non-Hispanic White (42%), followed by Hispanic/Latino (27%), African American (17%) and Chinese American (14%). On average, men were overweight with a mean BMI of 27.6 kg/m^2^. The participants reported an average of 12 h a week of physical activity. Moreover, 42% of participants were hypertensive, 13% stated active cigarette smoking, 15% had diabetes mellitus, and 24% were taking a cholesterol-lowering medication. The mean *total testosterone* level was 15 nmol/L.

**Table 1:** Baseline characteristics of the study population.

MEN (N = 878)	Mean ± SD/N (%)
Age (years)	61.6 (± 10.02)
SBP (mmHg)	125.7 (± 19.3)
DBP (mmHg)	75.4 (± 9.3)
Total Abdominal Muscle Area (cm^2^)	116.3 (± 24.1)
Total Abdominal Muscle Area Index (cm^2^/BMI)	4.26 (± 5.0)
Total Abdominal Muscle Radiodensity (HU)	44.4 (± 5.0)
Abdominal Stabilizing Muscle Area (cm^2^)	87.4 (± 20.2)
Abdominal Stabilizing Muscle Radiodensity (HU)	42.1 (± 5.4)
Abdominal Locomotor Muscle Area (cm^2^)	29.1 (± 6.1)
Abdominal Locomotor Muscle Radiodensity (HU)	51.3 (± 5.0)
Abdominal Visceral Fat Area (cm^2^)	163.0 (± 70.9)
Abdominal Subcutaneous Fat Area (cm^2^)	209.0 (± 92.7)
BMI (kg/m^2^)	27.6 (± 4.2)
WHR	0.96 (± 0.1)
hsCRP (mg/L)	2.5 (± 4.35)
TT (nmol/L)	15.0 (± 5.6)
fT (pmol/L)	300 (± 100)
SHBG (nmol/L)	43.4 (± 18.2)
Estradiol (nmol/L)	0.12 (± 0.0)
Race/Ethnicity	
Non-Hispanic White	368 (42%)
Chinese American	125 (14%)
Black	153 (17%)
Hispanic/Latino	229 (27%)
Time from baseline to CT (years)	2.63 (± 1.0)
Total Physical activity (hours/week)	11.99 (± 5.5)
Sedentary Behavior (hours/week)	24,8 (± 14.5)
Current cigarette smoker	115 (13%)
Diabetes Mellitus	129 (15%)
Hypertension	368 (42%)
Cholesterol medicine use	213 (24%)
Current alcohol use	577 (71%)
Drinks per week (current drinkers)	5.3 (± 7.2)

*SBP* systolic blood pressure, *DBP* diastolic blood pressure, *BMI* body mass index, *WHR* waist-hip-ratio, *hsCRP* high sensitivity c-reactive protein, *SHBG* sex-hormone binding globulin, *fT* free Testosterone, *TT* total testosterone.

### Association between sex hormones and abdominal muscle area

*Total* testosterone was significantly associated with total abdominal muscle area in the first model (B = 1.39, 95% CI 0.0–2.8, *p* = 0.05), which was accentuated with further adjustment (Model 2: 1.81, 0.2–3.5, *p* = 0.03; Model 3: 1.74, 0.1–3.3 *p* = 0.01) (Table 2). No significant associations were found between *total testosterone* and abdominal *stabilizing* muscle area (Table 3), while the associations were significant in all models for abdominal *locomotor* muscle area (Table 4).

**Table 2:** (a–c) Association between levels of serum testosterone (total and free), SHBG, estradiol and abdominal muscle variables.

Testosterone		fT		SHBG		ESTRADIOL	
B	95% CI	B	95% CI	B	95% CI	B	95% CI
(a) Total Abdominal Muscle Area							
Model 1							
1.39	− 0.0, 2.8	1.09	− 0.4, 2.5	0.24	− 1.3, 1.8	2.14	0.8,3.6
Model 2							
1.81	0.2, 3.5	1.11	− 0.3, 2.6	0.24	− 1.3, 1.8	1.97	0.6, 3.4
Model 3							
1.74	0.1, 3.4	1.10	− 0.3, 2.6	0.21	− 1.3, 1.8	1.84	0.4, 3.3
(b) Total Abdominal Muscle Area Index							
Model 1							
0.1	0.0, 0.2	0.1	− 0., 0.1	0.0	0.0, 0.1	0.10	0.0, 0.1
Model 2							
0.1	0.1, 0.2	0.1	0.0, 0.1	0.0	0.0, 0.1	0.05	0.0, 0.1
Model 3							
0.1	0.1, 0.2	0.1	0.0, 0.1	0.0	0.0, 0.1	0.05	0.0, 0.1
(c) Total Abdominal Muscle Radiodesity							
Model 1							
0.04	− 0.2, 0.3	0.16	− 0.1, 0.4	− 0.35	− 0.6, − 0.1	0.17	− 0.1, 0.4
Model 2							
0.32	0.1, 0.7	0.19	− 0.1, 0.5	− 0.35	− 0.6, − 0.1	0.16	− 0.1, 0.4
Model 3							
0.32	0.1, 0.6	0.21	− 0.1, 0.5	− 0.33	− 0.6, − 0.1	0.14	− 0.1, 0.4

Linear regressions are used to investigate the associations in three models. Model 1(adjustment for age, race/ethnicity, and visceral adipose tissue), model 2(adjustment for model 1 + SHBG, CRP, physical activity, sedentary behavior, cigarette smoking, alcohol use and time from baseline to CT), model 3 (adjustment for model 2 + hypertension, diabetes mellitus, dyslipidemia, cholesterol medication, thyroid agents, oral steroid use), SHBG (sex-hormone binding globulin), CRP (C-reactive protein), fT (free testosterone).

**Table 3:** (a–c) Association between testosterone (total and free), SHBG, estradiol and abdominal stabilizing muscles.

Testosterone		fT		SHBG		ESTRADIOL	
B	95% CI	B	95% CI	B	95% CI	B	95% CI
(a) Abdominal Stabilizing Muscle Area							
Model 1							
0.87	− 0.35, 2.1	0.71	− 0.5, 1.95	0.07	− 1.2, 1.38	1.45	0.2, 2.6
Model 2							
1.16	− 0.3, 2.6	0.74	− 0.5, 2.0	0.06	− 1.3, 1.4	1.30	0.1, 2.5
Model 3							
1.21	− 0.2, 2.6	0.76	− 0.5, 2.0	− 0.06	− 1.4, 1.3	1.16	0.0, 2.4
(b) Abdominal Stabilizing Muscle Area Index							
Model 1							
0.10	0.0, 0.1	0.0	− 0.0, 0.1	0.3	0.0, 0.1	0.04	0.0, 0.1
Model 2							
0.07	0.0, 0.1	0.0	0.0, 0.1	0.03	0.0, 0.1	0.03	0.0, 0.10
Model 3							
0.10	0.0, 0.1	0.1	0.0, 0.1	0.02	0.0, 0.1	0.03	0.0, 0.10
(c) Abdominal Stabilizing Muscle Radiodensity							
Model 1							
0.08	− 0.2, 0.4	0.20	− 0.1, 0.5	− 0.31	− 0.6, 0.0	0.14	− 0.2, 0.4
Model 2							
0.36	0.2, 0.7	0.23	− 0.1, 0.5	− 0.31	− 0.6, 0.0	0.11	− 0.2, 0.4
Model 3							
0.35	0.1, 0.7	0.24	− 0.0, 0.6	− 0.29	− 0.6, 0.0	0.10	− 0.2, 0.4

Linear regressions are used to investigate the associations in three models. Model 1(adjustment for age, race/ethnicity, and visceral adipose tissue), model 2(adjustment for model 1 + SHBG, CRP, physical activity, sedentary behavior, cigarette smoking, alcohol use and time from baseline to CT), model 3 (adjustment for model 2 + hypertension, diabetes mellitus, dyslipidemia, cholesterol medication, thyroid agents, oral steroid use), SHBG (sex-hormone binding globulin), CRP (C-reactive protein), fT (free testosterone).

**Table 4:** (a–c) Association between testosterone (total and free), SHBG, estradiol and abdominal locomotor muscles.

Testosterone		fT		SHBG		ESTRADIOL	
B	95% CI	B	95% CI	B	95% CI	B	95% CI
(a) Abdominal Locomotor Muscle Area							
Model 1							
0.52	0.2, 0.9	0.40	0.0, 0.7	0.17	− 0.2, 0.6	0.70	0.3, 1.1
Model 2							
0.65	0.2, 1.1	0.40	0.0, 0.7	0.18	− 0.2, 0.6	0.67	0.3, 1.0
Model 3							
0.63	0.2, 1.0	0.34	− 0.0, 0.7	0.14	− 0.3, 0.5	0.69	0.3, 1.0
(b) Abdominal Locomotor Muscle Area Index							
Model 1							
0.05	0.0, 0.1	0.0	0.0, 0.0	0.02	0.0, 0.0	0.02	0.0, 0.1
Model 2							
0.10	0.0, 0.1	0.0	0.0, 0.0	0.02	0.0, 0.0	0.02	0.0, 0.1
Model 3							
0.10	0.0, 0.1	0.0	0.0, 0.0	0.01	− 0.0, 0.0	0.02	0.0, 0.1
(c) Abdominal Locomotor Muscle Radiodensity							
Model 1							
− 0.1	− 0.4, 0.2	0.52	0.2, 0.9	− 0.5	− 0.8, − 0.2	0.27	− 0.0, 0.6
Model 2							
0.23	− 0.1, 0.6	0.1	− 0.3, 0.4	− 0.5	− 0.8, − 0.2	0.28	− 0.0, 0.6
Model 3							
0.23	− 0.2, 0.6	0.1	− 0.2, 0.4	− 0.5	− 0.8, − 0.2	0.26	− 0.0, 0.6

Levels of estradiol were significantly associated with *total* abdominal muscle area in all three models: Model 1 (2.14, 0.8–3.6, *p* < 0.01), Model 2 (1.97, 0.6–3.4, *p* < 0.01), Model 3 (1.84, 0.4–3.3, *p* = 0.01) with similar results for abdominal locomotor and abdominal stabilizing muscle area (Tables 3 and 4).

No significant associations were found between levels of *free* testosteron*e* and *total* abdominal muscle areas (Table 2), as well as stabilizing. However, significant associations were presented between *free* testosterone and locomotor area in model 1 & model 2(0.38, 0.0–0.7, *p* = 0.04, 0.37, 0.0–0.7, *p* = 0.04) with borderline significance in model 3 (0.34, − 0.0, 0.7, *p* = 0.05), respectively (Tables 2, 3, 4). Positive, although non-significant associations were found for SHBG with abdominal muscle areas (Tables 2, 3, 4).

### Associations between sex hormones and abdominal muscle radiodensities

*Total* testosterone was significantly associated with total abdominal muscle radiodensity in Models 2 and 3, but not in Model 1 (Model 1: 0.04, − 0.2 to 0.3, *p* = 0.79; Model 2; 0.32, 0.1–0.7, *p* = 0.04; Model 3: 0.34, 0.0–0.6, *p* = 0.04) (Table 2). Similar results were found for radiodensity of stabilizing muscles (Table 3), but not for locomotor muscle (Table 4).

No significant associations were found between *free* testosterone and abdominal muscle radiodensities in fully adjusted models (Tables 2, 3, 4).

No significant associations were found between levels of estradiol and total abdominal and stabilizing muscle radiodensity (Tables 2, 3), but there was a borderline significant association between estradiol and abdominal locomotor muscle radiodensity ((Model 1: 0.27, − 0.0 to 0.6, *p* = 0.09: Model 2: 0.28, − 0.0 to 0.6, *p* = 0.07; Model 3: 0.26, − 0.0 to 0.6, *p* = 0.09) (Table 4).

Higher SHBG levels were associated with a lower radiodensity of abdominal muscle in all models (Model 1: − 0.35, − 0.6 to − 0.1, *p* = 0.02: Model 2: − 0.35, − 0.6 to − 0.1, *p* = 0.02; Model 3: − 0.34, − 0.6 to − 0.1, *p* = 0.02) (Table 2). The results were similar for abdominal stabilizing and locomotor muscles (Table 3).

### Associations between sex hormones and abdominal muscle area indexes

A significant association was found in all models for *total* testosterone and TAMAi (Model 1: B = 0.10, 0.0–0.2, *p* < 0.01, Model 2: 0.11, 0.1–0.2, *p* < 0.01, Model 3: 0.10, 0.1–0.2, *p* < 0.01) (Table 2). That is, in fully adjusted models, one SD increase in testosterone levels resulted with an increase of 0.10 cm^2^/(weight/height^2^) in abdominal muscle area index. Similar relationships were observed between *total* testosteron*e* and abdominal locomotor and stabilizing muscle area indices (Tables 3, 4).

Estradiol was found to be significantly associated with total abdominal muscle index (TAMAi) in model 1 (B = 0.10, 0.0–0.1, *p* = 0.03), which was borderline significant in Models 2 and 3 (Model 2: 0.05, − 0.0 to 0.1, *p* = 0.06, Model 3: 0.05, − 0.0 to 0.1, *p* = 0.06). Significant associations were found for estradiol and abdominal locomotor muscle area index in all three models but not for abdominal stabilizing muscle area index (Tables 3 and 4).

Significant associations were shown for free testosterone with total, stabilizing and abdominal muscle area index in fully adjusted models (0.08, 0.0, 0.1, *p* = 0.008, 0.05, 0.0–0.1, *p* = 0.03, 0.02, 0.0–0.04, *p* = 0.02), respectively. Non-significant associations were found between levels of SHBG and abdominal muscle area indexes in both models 2 and 3 (Tables 2, 3, 4).

Significant negative correlations were presented between visceral fat and total testosterone (r = − 0.22, *p* < 0.001), and total abdominal muscle radiodensity (r = − 0.40, *p* < 0.001), and SHBG (− 0.18, *p* < 0.001), while a positive association was found for total abdominal muscle area (r = 0.11, *p* < 0.001) and estradiol (0.02, *p* = 0.6). Furthermore, the association between visceral fat and abdominal muscle area and radiodensity, significant associations were found (B = 0.03 *p* = 0.011, B = − 0.03 *p* < 0.001, respectively) even after adjustment for sex hormones.

## Discussion

Our study presents novel findings on the associations between sex hormones and SHBG and abdominal muscles in men. First, our results indicate that increases in serum levels of total testosterone and estradiol were associated with significant increases in abdominal muscle mass in men. In fact, our data indicate that the associations were stronger for estradiol than total testosterone with abdominal muscle mass to include both stabilizing and locomotor muscles. Second, significant associations were found between higher levels of total testosterone and abdominal muscle radiodensities. Third, our study presented a significant negative association between SHBG and abdominal muscle radiodensity.

A significant association was found between *total* testosterone and total abdominal muscle area in all models, with similar associations presented for abdominal locomotor muscle area but not for abdominal stabilizing muscle area. A plausible explanation of these differences might be that locomotor muscle contains a greater number of types II myofibers, a more dynamic and power related muscle type^11^. Type I myofibrils, which are predominantly found in the abdominal stabilizing muscles, have shown to be rather associated with endurance and higher lipid content^11–14^. Even though some studies have shown that supraphysiologic levels of testosterone increase both type I and II myofibers equally, other studies have reported testosterone affects maximal voluntary strength rather than endurance^5,15^. Another potential explanation to different associations between total testosterone and abdominal muscle groups could be from earlier studies, observing that administration of testosterone caused dose-dependent and region-specific changes in muscle mass^16^. This could potentially indicate that testosterone levels act differently strong, dependent on body region, type of muscle fibers and proportion of ectopic fat.

Interestingly, when adjustment was made for SHBG, lifestyle factors and time from baseline to CT in Model 2, a negative confounding was observed between *total* testosterone and both *total* abdominal muscle area and radiodensity. This underscores the significance of implementing additional adjustment, as the presence of visceral fat may obscure the true relationship between sex hormones and muscle composition. Furthermore, when adjustments were made for cardiometabolic disorders, similar associations were found. This could potentially suggest that the observed associations were less influenced by underlying metabolic conditions or were masked by prior adjustments.

Free testosterone was positively associated with total abdominal muscle area and radiodensity although, significance was found for locomotor muscle area in model 1 and 2 with borderline significance in model 3. *Total* and *free* testosterone was positively associated with TAMAi, abdominal stabilizing and locomotor muscle area indexes. In concurrence with our findings, Han et al. reported similar outcomes presented between *total* testosterone and abdominal muscle area index in men^17^. However, no adjustments were made for SHBG.

In our study, *total* testosterone was associated with increased total and stabilizing abdominal muscle radiodensities, independent of confounding factors.

Our results suggest that *total* testosterone is significantly associated with the degree of radiodensity of abdominal muscles, including muscle size. *Total* testosterone mainly includes SHBG-bound testosterone which has long been assumed to be inactive. However, recent experimental studies have shown the endocytic Megalin receptor, found in human skeletal myocytes, transports SHBG-bound testosterone and estradiol into cells^18,19^. This could indicate *total testosterone* may have an active role in cell regulation and muscle activity.

Our study found that higher levels of estradiol were significantly associated with higher levels of all abdominal muscle areas. In men, a great quantity of estradiol originates from the aromatase conversion of testosterone, which correlates with fat mass, activating estradiol receptors (ERs) in muscles^20,21^. This potentially suggests that testosterones` impact on muscles partly operates indirectly through its conversion to estradiol. Estradiol has earlier been found to play a key role in regulation of myokines, i.e., skeletal muscle proteins, with critical functions associated with exercise-related benefits and inflammation regulation in tissues^22^. Similarly, in a study on elderly Swedish men, estradiol, and not testosterone, was associated with lean mass measured with DXA scans^9^.

Estradiol was positively but non-significantly associated with total and stabilizing muscle radiodensities, while a borderline-significant association was shown with locomotor muscle radiodensity. Studies have reported higher concentrations of estradiol receptors in skeletal muscles of men engaged in greater endurance trainings as well as that supplementation of estradiol increases lipid utilization in skeletal muscles, increasing strength^23,24^. Whether there is region-specific dose-dependent effects is unknown. However, differences in associations between different sex hormone concentrations and various muscle radiodensities may stem from distinct mechanisms of action mediated by activation of androgen receptors or estrogen receptors in skeletal muscles. It has been demonstrated in animal experiments, that these receptors activate different genes, potentially accounting for the observed differences^25^.

The bioactive role of SHBG is still debated. An inverse association has been shown between SHBG levels and insulin resistance and metabolic syndrome^48^. Other studies have found a positive association between SHBG and inflammatory cytokines, low protein diet and hip fractures in elderly even after adjustment for sex hormones^26,27^. Our study showed an inverse association between SHBG and all abdominal muscle densities. Other results have reported an inverse relationship between SHBG and lean muscles measured by dual-energy x-ray absorptiometry (DXA) scans^9^. Furthermore, Yuki et al. reported SHBG levels were significantly higher in the group of individuals diagnosed with sarcopenia compared to the normal group^28^. In agreement with our findings, SHBG has been reported to have a significant inverse association with muscle strength in elderly men^29^. One plausible cause of the negative associations between SHBG and abdominal muscle radiodensities could be that an increase in SHBG concentrations may influence the binding capacity and magnitude of available free testosterone and could suggest a partial explanation to some of the weaker association found for other sex hormones^30^.

We imply the importance of investigating the interplay among sex hormones, SHBG and muscle tissue composition, given prior findings suggesting that both muscle area and radiodensity exert notable influences on survival among men^31^.

This study has a number of strengths, including usage of data from a large and diverse cohort, detailed sampling of information with validated instruments as well as standardized sampling of blood specimens according to guidelines^32^. Furthermore, by assessing muscle composition with CT, we were able to indirectly estimate its quality. In earlier studies, radiological slices of the lumbar region are approved when assessment of total muscle volumes are made^33^. However, our study does have some limitations. First, radioimmunoassay technique (RIA) was used to measure sex hormones and SHBG. This has been described to be less precise than mass spectrometry in the measurement of sex-hormone levels^34^. Studies have found that it could potentially result in difficulties in distinguishing eugonadal from mildly hypogonadal males^35^. Furthermore, levels of sex hormones may be affected by the presence of several cross-reacting steroids^36^. Second, *free* testosterone was calculated and not directly measured which has been shown to overestimate levels compared with laboratory measured free testosteronex^37^. While the Sodergard method has previously been described as one of the most common methods for calculating free testosterone in endocrinology literature, it has limitations, including a predefined albumin concentration, higher estimates compared to other algorithms and accurate mainly when competing steroids to binding sites are limited and normal levels of SHBG are involved^38,39^. Furthermore, the Sodergard method presents concordant results to the Vermuelen algorithm and its association constant was validated when compared to results of calculations with a gold standard technique^38^. Third, the sex hormones were measured at visit 1 whilst CT scans were made at visit 2 and visit 3. We partially addressed this limitation by adjusting for the time from baseline to CT scan as a confounder in model 2 and model 3. Also, the energy levels of the X-ray beams differed between the CT-scans (120–140 kVp) which is a further limitation. In addition, measurements of physical activity and sedentary behavior were self-reported.

We only evaluated abdominal muscle area and radiodensity, and therefore, our findings may not be applicable to peripheral muscles. Finally, this study design was observational and cross-sectional, which is prone to residual confounding, as well as temporal and selection biases.

## Conclusion

In this analysis, we demonstrate a positive association between total testosterone levels with abdominal muscle area and radiodensity, whereas estradiol showed a similar strong association with abdominal muscle area but not radiodensity. Additionally, SHBG was significantly and inversely associated with abdominal radiodensity. These results suggest the relevance of sex hormone levels to maintain muscle mass and density with advancing age.

## Material and methods

### Study design and study population

At baseline (2000 to 2002), 6814 adult men and women between 45–84 years that were free of clinical cardiovascular disease were recruited into the Multi-Ethnic Study of Atherosclerosis (MESA). Participants were enrolled from six US communities (New York [NY], Baltimore [MD], Chicago [IL], Los Angeles [CA], Twin Cities [MI] and Winston-Salem [NC]). Approximately 38% were Non-Hispanic White, 28% African American, 23% Hispanic American, and 11% Chinese American.

### Data collection

Details on the MESA cohort methods have been published^40^. Briefly, trained staff performed specimen blood draws and processing of venous blood samples, blood pressure measurements and all interviews. Using standard procedures, fasting blood samples were processed and stored at − 80 °C^41^.

Information on lifestyle factors, medications and co-morbidities were gathered using validated questionnaires. Race/ethnicity was self-reported at baseline according to 2000 US Census criteria. All individuals treated with sex hormones at baseline were excluded from the study. Physical activity (hours/week) and sedentary behavior (hours/week) were measured by using a comprehensive, semiquantitative questionnaire^42^. Current medication use was assessed according to standardized questionnaires^43^. Hypertension was defined as a systolic blood pressure above 140 mmHg and/or a diastolic above 90 mmHg or taking a blood pressure lowering medication. Diabetes mellitus was defined as self-reported diabetes, fasting glucose levels according to the American Diabetes Association or use of glucose lowering medications^44,45^. Measurement of high-sensitivity C-reactive protein (hsCRP), a marker of systemic inflammation, has previously been described^41,46^.

### Computed tomography for body composition

A random subset of 1970 participants (946 men) at visits 2 and 3 (2002 to 2005) were enrolled in an ancillary study obtaining abdominal computed tomography (CT) scans. The original aim of investigating CT-scans of the abdominal cavity was to measure the abdominal aortic calcium. Recently, approved data from the CT-scans were used to assess information on muscle composition such as muscle radiodensity and muscle area, which were then interrogated for abdominal muscle area, abdominal radiodensity, visceral adipose tissue and subcutaneous fat tissue. At two clinical sites (Northwestern University, University of California Los Angeles) electron-beam CT scanner (Imatron C-150) was used while at the remaining clinical sites (Columbia University, Wake Forest University, and University of Minnesota) multi-detector CT scanners (Sensation 64 GE lightspeed, Siemens S4 Volume Zoom, and Siemens Sensation 16) were used. A 35 cm field of view was used. In cases where a cut off was seen, imputation methods such as doubled values with repeat measure t-tests on a random sample, regression equations and ruler lines were used to estimate the areas. CT scans were set at a collimation of 3 mm with a slice thickness of 6 mm. In total, two cross-sectional slices were taken at L2/L3, L3/L4 and L4/L5 intervertebral disc spaces, constituting a total of six slices. Approximately half of the participants underwent CT scans at visit two and the other half at visit three.

**Figure 1:**
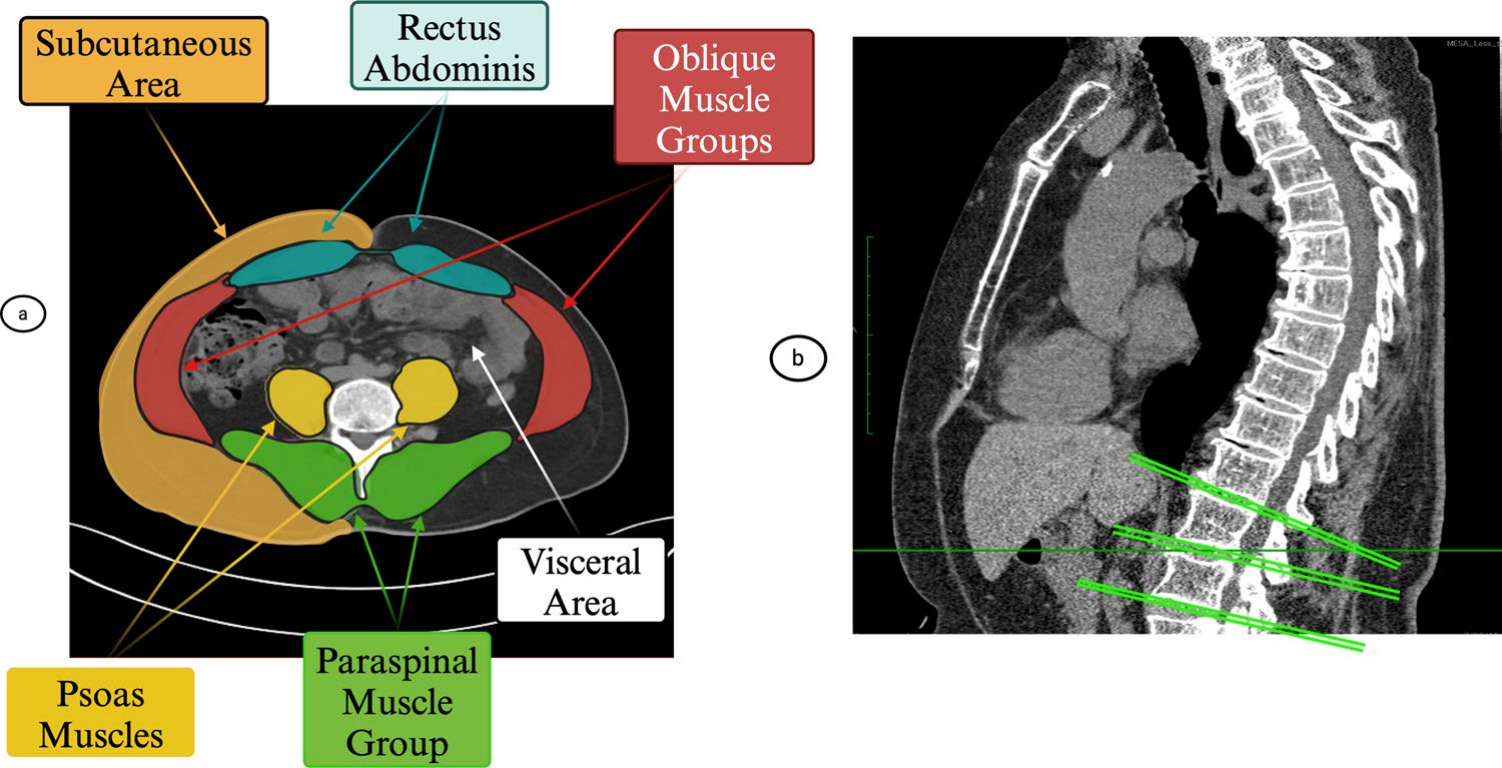
(**a**) Presents an axial slice from the lumbar region, including abdominal adipose and muscle tissue. Abdominal tissue was categorized by Hounsfield units (HU), with − 190 to − 30 HU assessed as adipose tissue, − 30 to − 0 HU defined as mixed connective tissue, while values 0 to 100 HU were set as lean muscle^47,48^. Abdominal muscle area and adipose tissue area were calculated by summing the number of pixels, while muscle radiodensity was defined by average HU value measured within that muscle’s corresponding fascial plane. The abdominal muscles were further categorized into stabilizing muscle groups (rectus abdominis, oblique muscle groups and paraspinal muscles) and locomotor muscle groups (psoas muscles). Subcutaneous adipose tissue was determined as adipose tissue in the subcutaneous area, whilst visceral adipose tissue was determined as fat tissue in the visceral cavity, excluding intermuscular fat. (**b**) Presents a sagittal slice from the lumbar region. 6 transverse cross section slices of data were analyzed; slice 0 is located at the L4/L5 vertebral junction and slice 1 is the immediately superior and adjacent to slice 0. Slice 2 is located at the L3/L4 junction with slice 3 superior and adjacent to slice 2. Slice 4 is located at the L2/L3 vertebral junction with slice 5 superior and adjacent to slice 4. CT scans were set at a collimation of 3 mm with a slice thickness of 6 mm.

Assessment of abdominal muscles and adipose tissue were obtained from these CT scans (Fig. 1a, b) and have been described earlier^31^. Using a semi-automated method, measurement of total tissue, lean muscle, and adipose tissue were assessed in each slice using Medical Imaging Processing Analysis and Visualization (MIPAV) software version 4.1.2 (National Institutes of Health, Bethesda, Maryland). Abdominal tissue was categorized by Hounsfield units (HU) with − 190 to − 30 HU assessed as adipose tissue, − 30 to − 0 HU defined as mixed connective tissue or undefined, while values 0 to 100 HU were set as muscle tissue^47,48^. Abdominal muscle area and adipose tissue area were calculated by summing the number of pixels while muscle radiodensity was defined by average HU value measured within that muscle’s corresponding fascial plane. Research staff responsible for analyzing CT scans were blinded to participants’ clinical information. The inter- and intra-rater reliability of measurements for total abdominal area as well as measurements for all muscle groups was 0.99 and 0.93 to 0.98, respectively^28^.

Visceral adipose tissue was determined as fat tissue in the visceral cavity, excluding intermuscular fat. Four abdominal muscle groups were assessed bilaterally. Area and radiodensity of the oblique and paraspinal muscle group, and rectus abdominis comprised the muscles of stabilization, while the psoas muscles were the locomotor group. Combined area and radiodensity of muscles of stabilization and locomotion were defined as total abdominal muscle area (TAMA) and radiodensity (TAMD), respectively.

Adjustment for body mass index (BMI, kg/m^2^) was made for abdominal muscle areas and were defined as abdominal muscle indexes (total abdominal muscle area index (TAMAi) (TAMAi = TAMA/BMI), stabilizing muscle area index (TSMA/BMI) and locomotor muscle area index (TLMA/BMI)^17^.

**Figure 2:**
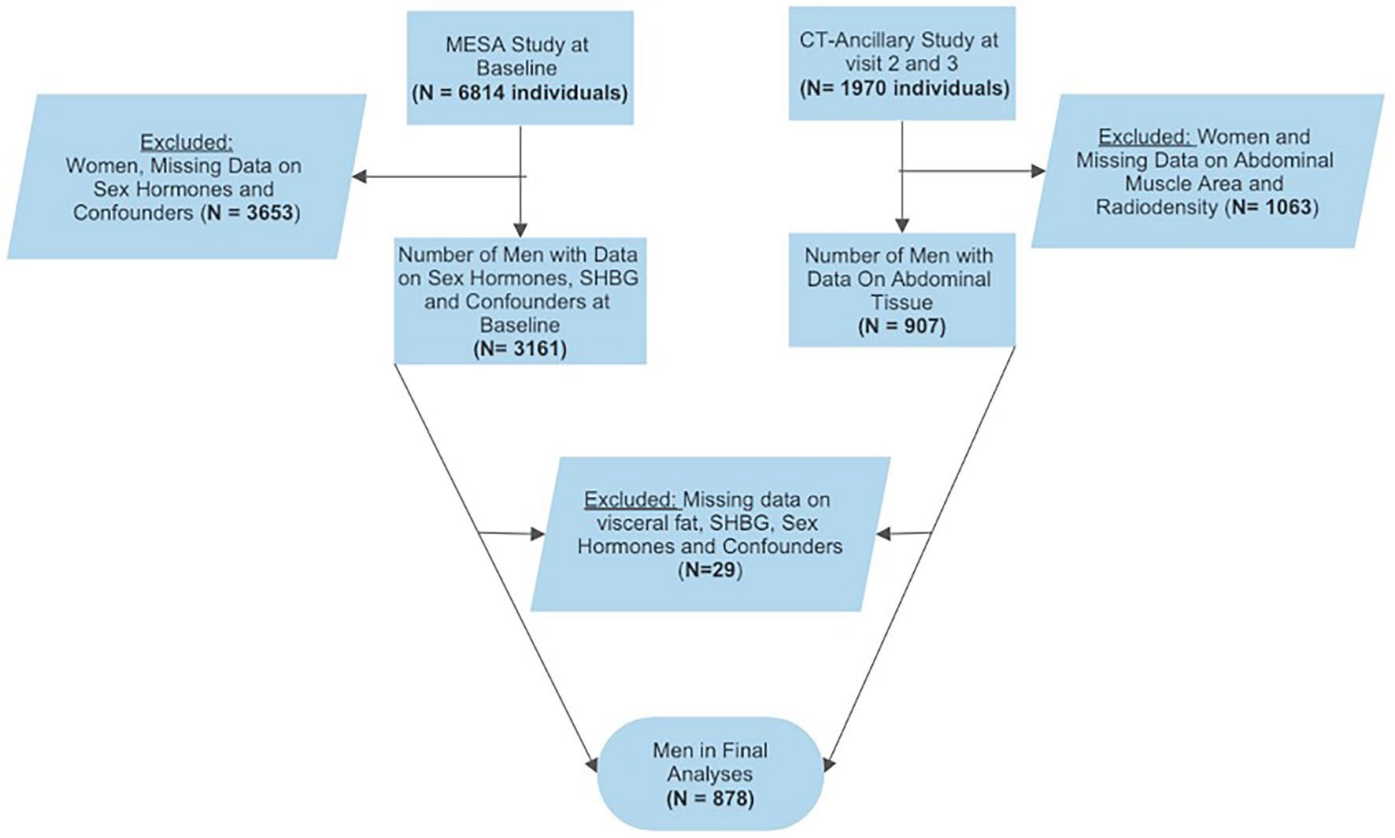
Flow-chart showing the number of men included in the final analyses.

For this analysis we excluded participants with missing data from CT scans, anthropometric measurements, endogenous sex hormones, lifestyle factors, co-morbidities, and medication use. The final sample size included 878 men (Fig. 2).

### Assessment of endogenous sex hormones

Measurement of endogenous sex hormone levels have previously been described^49,50^. In brief, total testosterone was measured using radioimmunoassay kits. Sex hormone binding globulin (SHBG) concentration was assessed by Chemiluminescent enzyme immunometric assay (Immulite kits, Diagnostic Products Corporation, Los Angeles, CA). A ~ 10% blind pool was obtained to assess quality control serum. The coefficients of variation for total testosterone and SHBG were 12.3% and 9.0%, respectively^51^. Estradiol was measured using an ultra-sensitive radioimmunoassay kit (Diagnostic System Laboratories, Webster, TX) with a coefficient of variation of 10.5%^51^. Free testosterone( nmol/L) was calculated according to the method of Södergård^52^.

### Statistical analysis

Continuous variables were reported as means and standard deviations (SD) while categorical variables were shown as frequencies and percentages. Abdominal muscle areas/radiodensities/indexes were the outcome variables. Total, locomotor and stabilizing abdominal muscles showed normal distributions. As such, multivariable linear regression models were conducted to assess the associations between levels of sex hormones and abdominal muscles. The outcome was defined as the change in HU for abdominal muscle radiodensity and square centimeters for abdominal muscle area for each 1-SD increment change in levels of testosterone, estradiol and SHBG.

Model 1 adjusted for age, race/ethnicity, and visceral adipose tissue. Model 2 included variables from model 1 and SHBG (when investigating the associations of total testosterone and estradiol), CRP, physical activity, sedentary behavior, cigarette smoking, alcohol use, and time from baseline to CT. Model 3 included variables from Model 2 with further adjustment for hypertension, diabetes mellitus, dyslipidemia, use of oral steroids or cholesterol/thyroid/hypertension/glucose lowering/ medication. No adjustment was made for SHBG when free testosterone was the independent variable.

A two-tailed *p*-value < 0.05 was considered as statistically significant. Analyses were conducted using IBM SPSS Statistics, version 29.

### Ethical considerations

The MESA study protocol was approved by the Institutional Review Board at the Johns Hopkins University Hospital, University of California Los Angeles, University of Minnesota, Wake Forest University Hospital, Northwestern University Hospital, and Columbia University. All methods were performed in accordance with the relevant guidelines and regulations as set by the approving institutions in a standardized manner. Written informed consent was given by all participants^40^.

## Data Availability

The supporting data for the conclusions drawn in this study can be obtained from the MESA committee, but access is subject to certain restrictions as they were utilized under license for the present study and are not publicly accessible. Nevertheless, interested parties can request access to the data directly from the authors, pending approval from the MESA committee. https://www.mesa-nhlbi.org.
