# Correction: Levels of Sex Hormones and Abdominal Muscle Composition in Men from The Multi-Ethnic Study of Atherosclerosis

**DOI:** 10.1038/s41598-026-51929-6

**Published:** 2026-06-03

**Authors:** Amar Osmancevic, Matthew Allison, Iva Miljkovic, Chantal A. Vella, Pamela Ouyang, Penelope Trimpou, Bledar Daka

**Affiliations:** 1 General Practice / Family Medicine, School of Public Health and Community Medicine, Institute of Medicine, Sahlgrenska Academy, University of Gothenburg, Gothenburg, Sweden; 2 Division of Preventive Medicine, School of Medicine, UC San Diego, San Diego, CA USA; 3 Department of Epidemiology, School of Public Health, University of Pittsburgh, Pittsburgh, PA USA; 4 Department of Movement Sciences, University of Idaho, Moscow, ID USA; 5 Institute for Clinical and Translational Research, Johns Hopkins University School of Medicine, Baltimore, MD USA; 6 Department of Endocrinology, Institute of Medicine, Sahlgrenska Academy, University of Gothenburg and Sahlgrenska University Hospital, Gothenburg, Sweden

Correction to: *Scientific Reports* 10.1038/s41598-024-66948-4, published online 12 July 2024

The original version of this Article contained an error in the Conclusion.

“Additionally, SHBG was significantly and inversely associated with abdominal radiodensity although a negative trend was presented for abdominal muscle index.”

now reads:

“Additionally, SHBG was significantly and inversely associated with abdominal radiodensity.”

The original Article has been corrected.

